# Exploring the Therapeutic Potential of Extracellular Vesicles Derived from Human Immature Dental Pulp Cells on Papillary Thyroid Cancer

**DOI:** 10.3390/ijms25158178

**Published:** 2024-07-26

**Authors:** Michelli Ramires Teixeira, Anderson Lucas Alievi, Vitor Rodrigues da Costa, Irina Kerkis, Rodrigo Pinheiro Araldi

**Affiliations:** 1 Postgraduate Program in Endocrinology and Metabology, Escola Paulista de Medicina of the Universidade Federal de São Paulo (EPM/UNIFESP), São Paulo 04022-001, SP, Brazil; mr.teixeira@unifesp.br (M.R.T.); anderson.alievi@butantan.gov.br (A.L.A.); 2 Genetics Laboratory, Instituto Butantan, São Paulo 05503-100, SP, Brazil; vitor.rodrigues@unifesp.br (V.R.d.C.); irina.kerkis@butantan.gov.br (I.K.); 3 Postgraduate Program in Structural and Functional Biology, Escola Paulista de Medicina of the Universidade Federal de São Paulo (EPM/UNIFESP), São Paulo 04023-062, SP, Brazil; 4 BioDecision Analytics Ltda., São Paulo 05713-510, SP, Brazil

**Keywords:** papillary thyroid cancer (PTC), extracellular vesicles (EVs), cell-free therapy, human immature dental pulp stem cells (hIDPSCs), DPSCs

## Abstract

Mesenchymal stem-cell-derived extracellular vesicles (MSC-EVs) have been increasingly investigated for cancer therapy and drug delivery, and they offer an advanced cell-free therapeutic option. However, their overall effects and efficacy depend on various factors, including the MSC source and cargo content. In this study, we isolated EVs from the conditioned medium of human immature dental pulp stem cells (hIDPSC-EVs) and investigated their effects on two papillary thyroid cancer (PTC) cell lines (BCPAP and TPC1). We observed efficient uptake of hIDPSC-EVs by both PTC cell lines, with a notable impact on gene regulation, particularly in the Wnt signaling pathway in BCPAP cells. However, no significant effects on cell proliferation were observed. Conversely, hIDPSC-EVs significantly reduced the invasive capacity of both PTC cell lines after 120 h of treatment. These in vitro findings suggest the therapeutic potential of hIDPSC-EVs in cancer management and emphasize the need for further research to develop novel and effective treatment strategies. Furthermore, the successful internalization of hIDPSC-EVs by PTC cell lines underscores their potential use as nanocarriers for anti-cancer agents.

## 1. Introduction

Thyroid cancer (TC) is the most common endocrine malignancy [1,2]. The rise in new cases of TC is predominantly seen in one specific histological type, papillary thyroid carcinoma (PTC), which accounts for 90% of TC cases [3]. Although PTC generally has a favorable prognosis, it includes several subtypes that may pose a higher risk of metastasis and increased mortality [4,5,6]. In such cases, standard treatments, such as lobectomy, thyroidectomy, and radioactive iodine therapy, might not be effective, requiring more effective therapeutic approaches for patients with local and distant cancer cell dissemination [6,7].

In this regard, isolated and in vitro cultured mesenchymal/stromal stem cells (MSCs) have shown interesting therapeutic potential for cancer treatment, as they can modulate the immune system, promote apoptosis in cancer cells, disrupt neovascularization, and secrete trophic factors [8,9,10]. However, these MSCs have also been reported to promote cancer progression, including increased tumor proliferation, migration, invasion, metastasis, and drug resistance [10,11]. The mechanisms underlying the effects of MSCs on cancer are poorly understood, although increasing evidence suggests that these effects are, in part, related to cell communication mediated through soluble factors, particularly those released by cells and encapsulated by a lipid bilayer, known as extracellular vesicles (EVs) [12,13,14].

In the context of cancer, there is a special concern that crosstalk between MSCs and the tumor microenvironment could influence MSCs, potentially causing them to acquire pro-tumor behavior [9,15]. To address this issue, EVs derived from MSCs (MSC-EVs) cultured in vitro may offer a viable solution [16]. These specialized cell communication particles transport and deliver a wide range of biomolecules, such as RNAs (mRNA, microRNA, and other non-coding RNAs) and proteins, reflecting the physiological state and signature of their source cells [9,15]. 

Although reports have also shown a dual effect of different sources of MSC-EVs in various cancer models, EVs maintain their content and are not reprogrammed by the tumor microenvironment, as MSCs are believed to be [13]. This stability could reduce unexpected risks while retaining benefits, offering a more predictable biological tool that encompasses the paracrine therapeutic features of MSCs [8,17,18]. Additionally, MSC-EVs enable cell-free therapy, which offers advantages, such as low immunogenicity, small size, longer shelf life, easier storage, and the ability to nano-deliver specific chemotherapeutic molecules [19,20,21].

Therefore, we investigated the effects of EVs derived from human immature dental pulp stem cells (hIDPSCs) in two PTC cell lines harboring two distinct and the most common driver mutations in the disease: the *BRAFV600E* mutation (BCPAP) and *RET/PTC* rearrangement (TPC1) [22]. hIDPSCs, a promising type of MSCs found in the pulp tissue of teeth, have demonstrated unique therapeutic potential, particularly for neurodegenerative diseases [23,24,25]. This potential has led to their investigation in regulatory-approved clinical trials, with early findings suggesting that they can be safely utilized [23,24,25,26]. However, EVs derived from hIDPSCs have not gained significant attention and represent an unexplored area in biomedical applications, including cancer.

## 2. Results

### 2.1. hIDPSC-EVs’ Size Distribution and Internalization

The hIDPSC-EVs’ size distribution analysis using the NanoSight NS300 equipment showed a concentration of 1.10 × 10^9^ ± 0.13 × 10^9^ particles/mL, with a medium particle size of 210.2 ± 5.6 nm (Supplementary Figures S1 and S2). Protein measurements using the BCA method showed a medium concentration of 662.68 ± 5.17 μg/mL. 

**Figure 1:**
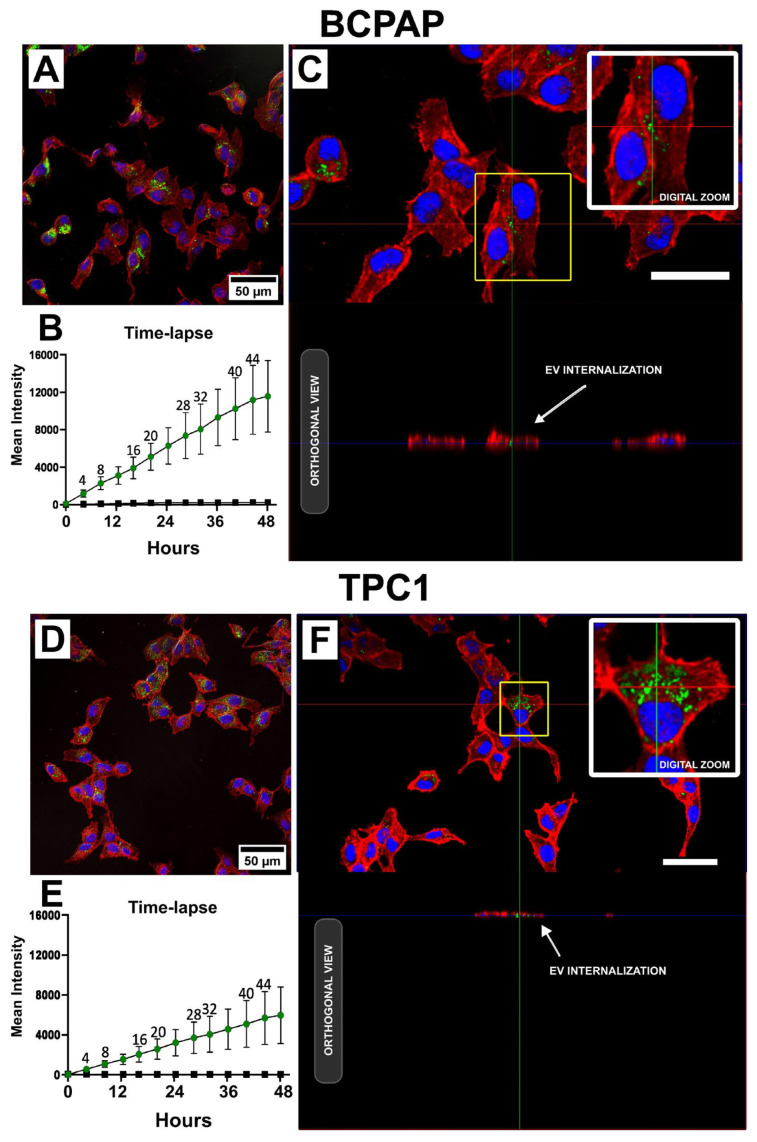
Uptake of hIDPSC-EVs by BCPAP and TPC1 cells. Figures (**A**,**C**,**D**,**F**) show representative confocal microscopy images of both cell lines treated with 50 μg/mL EVs-DiO (green) for 24 h. Cells were counterstained with Alexa Fluor 647 phalloidin (red) to determine the cytoskeleton and Hoechst 33342 (blue) to identify cell nuclei. Figures (**A**,**D**) show 2D images of perinuclear localization of EVs-DiO in BCPAP and TPC1 cells, respectively. Figures (**C**,**F**) show orthogonal views of the cell lines, with white arrows indicating the presence of EVs-DiO between cytoskeletal fibers (red), confirming EVs-DiO internalization. In (**C**,**F**), white bars represent a scale bar of 50 μm inserted using the ImageJ/FIJI software (version 1.54f). Figures (**B**,**E**) show the graphical results of time-lapse confocal microscopy, in which the internalization dynamics of EVs-DiO were investigated over 48 h with several images captured. The graphs show the mean fluorescence intensity (MFI) of EVs-DiO (curve with green dots) and Control-DiO (curve with black squares) internalized by BCPAP and TPC1 cells, respectively. Each point on the curve represents the mean of triplicate MFI measurements. Bars represent the standard deviation (SD). No statistical analyses were performed for this assay.

Uptake assays revealed the internalization of Vybrant DiO-labeled hIDPSC-EVs (EVs-DiO) in both BCPAP and TPC1 cell lines after 24 h of incubation, as shown in Figure 1. EVs-DiO were localized in the perinuclear region of the cells, with notably more efficient internalization observed in BCPAP cells. Time-lapse analysis further confirmed this difference in internalization efficiency between the two cell lines and demonstrated a time-dependent relationship with EV internalization. In contrast, Control-DiO did not exhibit time dependence or an increase in the mean fluorescence intensity over time, as shown in Figure 1B,E (Supplementary Videos S1 and S2; Supplementary Figures S3–S5). This indicated that the fluorescence observed in the assays was due to EVs and not micelles formed by the dye.

### 2.2. Transcriptional Analysis of Papillary Thyroid Carcinoma Cell Lines

**Figure 2:**
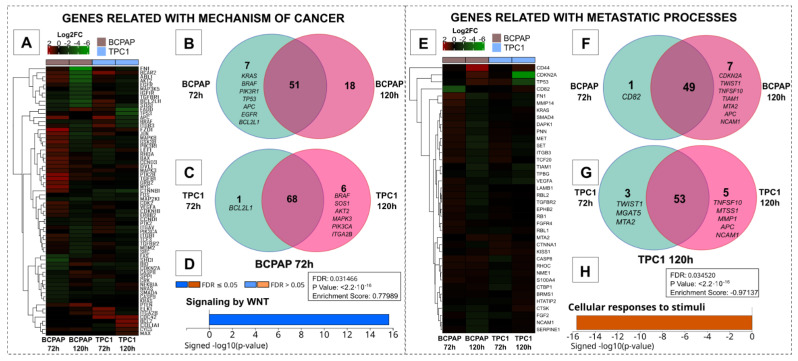
Representation of regulated genes in PTC cell lines after EVs treatment. Genes related to general mechanisms of cancer (left) showed pronounced regulation in both cell lines and a shift from a downregulated profile after 72 h of treatment to a more upregulated profile after 120 h of EV incubation (**A**). Besides the marked regulation, only the WNT pathway (**D**) showed significant regulation (*p* < 2.2 × 10^−16^; FDR: 0.031466), presented on the BCPAP 72 h post-treatment. The gene set regulated to metastatic processes (right) showed less pronounced gene regulation and only one significant pathway regulated (*p* < 2.2 × 10^−16^; FDR: 0.034520), in the TPC1 120 h post-treatment, named ‘Cellular responses to stimuli’ (**H**). Interestingly, few genes were exclusively regulated with the two periods of treatment analyzed in the same cell lines in both qPCR arrays (**B**,**C**,**F**,**G**). Heatmaps were generated using the Log2FC values of regulated genes and normalized to the untreated group (**A**,**E**).

Transcriptional analysis using a gene array focused on cancer mechanisms has revealed distinct patterns of gene regulation across different cell lines and treatment durations. The heatmap (Figure 2A) demonstrates that the BCPAP cell line exhibited more pronounced transcriptional changes than the TPC1 cell line. In both cell lines, most genes were downregulated at 72 h post-treatment. However, after 120 h, this trend was reversed, showing the upregulation of the majority of genes. Interestingly, only a few genes were exclusively affected within the treatments (Figure 2B,C). Despite these transcriptional alterations, significant enrichment was observed only in the Wingless Int-1 (Wnt) signaling pathway, after the 72 h treatment, in the BCPAP cell line, as analyzed using WebGestalt (*p* < 2.2 × 10^−16^; FDR: 0.031466). The participant genes enriched in this pathway included *FZD1*, *MYC*, *APC*, *GSK3B*, *LEF1*, *CTNNB1*, *DVL1*, *AKT2*, and *RHOA*, all of which were upregulated (Supplementary Figure S6). The complete gene set and their respective Log2FC are available in Supplementary Table S1. In the gene array focused on metastatic processes, a slight regulation of genes was observed compared to that in the previously analyzed array. Consistent with the previous array, the BCPAP cell line exhibited a shift from predominantly downregulated genes after 72 h of treatment to a more upregulated profile after 120 h of treatment. However, this pattern was not observed in the TPC1 cell line. Interestingly, despite the small number of regulated genes, only the TPC1 cell line showed a pathway with significant enrichment after 120 h of treatment (*p* < 2.2 × 10^−16^; FDR: 0.034520), namely the ‘Cellular responses to stimuli’ pathway, which comprised three regulated genes: *CDKN2A* (LogFC: −7.0309), *TP53* (LogFC: −3.2422), and *VEGFA* (LogFC: −1.1167) (Supplementary Figure S7). All the genes and their log2FC values are listed in Supplementary Table S2. The differential enrichment patterns suggest that the treatment has time-dependent and cell-line-specific effects. The BCPAP cell line responded more rapidly within 72 h, affecting the Wnt signaling pathway. In contrast, the TPC1 cell line showed significant changes after prolonged treatment (120 h) in a different cellular pathway. 

### 2.3. Functional Analysis of the Effects of hIDPSC-EVs on PTC

#### 2.3.1. hIDPSC-EVs Do Not Change the Proliferative Capacity of BCPAP and TPC1 Cells

**Figure 3:**
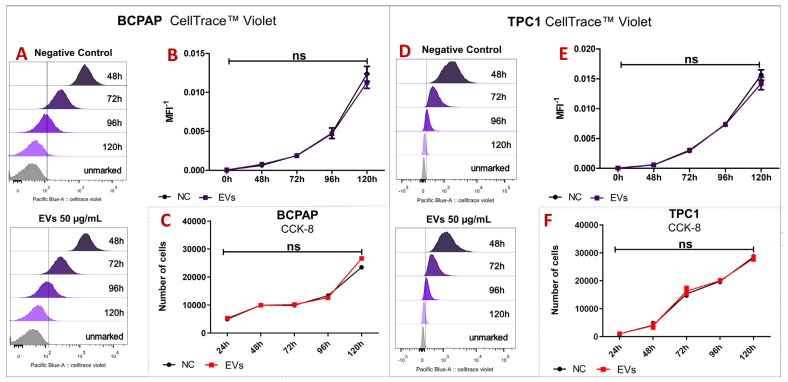
(**A**,**D**) display histograms of CellTrace-Violet-stained cells at four time points (48, 72, 96, and 120 h) along with unmarked control cells. No notable variance in cell proliferation was observed between the untreated (negative control) and treated (EVs) groups for either cell line. This finding was corroborated through statistical analysis (**B**,**E**) using the median fluorescence intensity (MFI). In the CCK-8 proliferation assay (**C**,**F**), cell counts were examined at five time points (24, 48, 72, 96, and 120 h), and there were no significant differences between untreated cells (NC: negative control) and treated cells (EVs). Each point on the curves represents the mean of triplicate MFI or the number of cell measurements. Bars represent the standard deviation (SD). Statistical analysis was performed using the median fluorescence intensity (MFI) at each time point (*n* = 3) analyzed using the CellTrace Violet assay (**B**,**E**) and the number of cells in the CCK-8 assay (**C**,**F**). The statistical test applied in both analyses was the Mann–Whitney test with a significance level of *p* < 0.05, conducted using GraphPad Prism 10. Statistical values with *p* > 0.05 are represented by “ns” (non-statistical).

CellTrace Violet and CCK-8 proliferation assays indicated that hIDPSC-EVs did not affect the proliferation of PTC cell lines for over 120 h. At each time point assessed, there were no statistically significant differences between the control and EV-treated groups. Notably, by the end of 120 h, neither group showed positive staining for CellTrace Violet, suggesting a reduction or cessation of cell proliferation (Figure 3).

#### 2.3.2. hIDPSC-EVs Do Not Affect BCPAP and TPC1 Cell Cycle, Viability, and Metabolism

**Figure 4:**
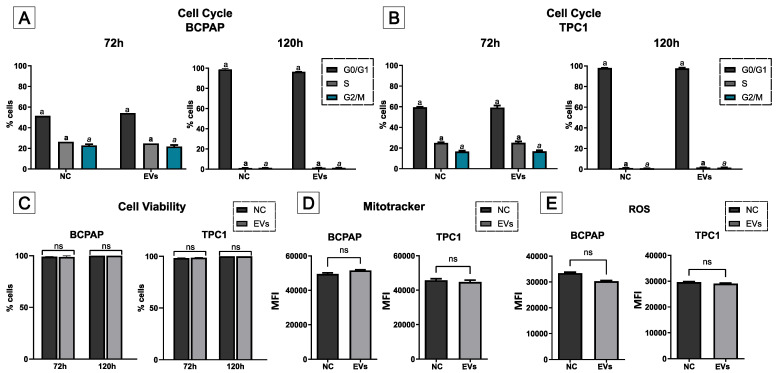
Functional assays using flow cytometry. Panels (**A**,**B**) show the cell cycle assays analyzed after 72 h (left graph) and 120 h (right graph) of EV treatment, with no statistically significant differences observed between the groups. Dark gray bars represent the percentage of cells in the G0/G1 phase, light gray bars represent cells in the S phase, and blue bars represent cells in the G2/M phase (*n* = 3). Each phase of the cell cycle was compared only with cells in the same phase and not across different phases. Statistical differences between groups are indicated by letters above the bars. Identical letters indicate no statistically significant differences. Different text formats (normal, bold, and italics) are used to distinguish each phase (a: G0/G1; **a**: S; *a*: G2/M). Panels (**C**–**E**) represent cell viability (% of live cells; *n* = 3), mitochondrial membrane potential (median fluorescence intensity (MFI); *n* = 3), and ROS production assay results (MFI; *n* = 3) in both cell lines, respectively, with no statistical differences observed. Metabolic assays (MitoTracker and ROS assays) were conducted after 72 h of treatment. All bars represent the media of three independent assays. The error bars represent the standard deviation (SD). The Mann–Whitney statistical test was applied to all assays, with a significance level of *p* < 0.05. ns = *p* > 0.05.

Cell cycle analysis also revealed a reduction in the proliferation of PTC cells after 120 h of cell culture, with nearly all cells arrested in the G0/G1 phase of the cell cycle (Figure 4A,B). However, no statistical differences were detected between the control and EV-treated cells at either 72 or 120 h, indicating that EV treatment did not significantly affect cell cycle progression. Additional assays further demonstrated that EVs did not influence cell viability, mitochondrial membrane potential, or the production of reactive oxygen species at either time point (Figure 4A,E). Thus, the overall data suggest that hIDPSC-EVs do not have a noticeable impact on cell behavior in the tested cell lines within the given period.

#### 2.3.3. hIDPSC-EVs Decrease Cell Motility in BCPAP and TPC1 Cell Lines

**Figure 5:**
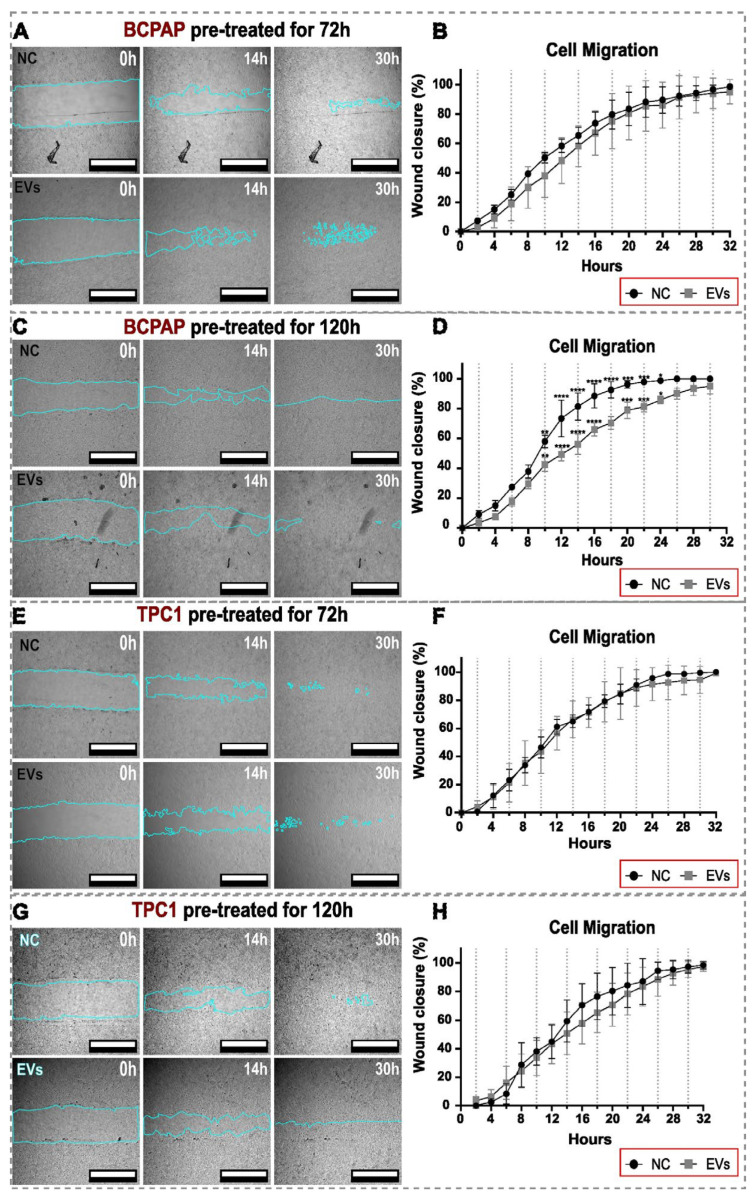
Migration of BCPAP and TPC1 cells. Figures (**A**,**C**,**E**,**G**) show representative images of the wound healing assay captured using confocal microscopy at three selected time points (0, 14, and 30 h) for both the negative control (NC) and EV groups. The cyan blue contour outlines the scratch limit of the area in ImageJ/FIJI software, which was used to analyze wound closure over time. The 32 h representative images were not used in the panel but were included in the statistical analysis because the wound was completely closed at this time point in almost all groups. In (**A**,**C**,**E**,**G**), white bars represent a scale bar of 800 μm inserted using the ImageJ/FIJI software. Graphics (**B**,**D**,**F**,**H**) show the wound area over time. The wound area was measured in μm^2^ and converted to the percentage of wound closure, with 0 h representing 0% wound closure. Images of the same scratch area were obtained every 2 h from 0 to 32 h of incubation. Each point on the curve represents the mean of triplicate MFI or the number of cell measurements. Bars represent the standard deviation (SD). Only the BCPAP 120 h group exhibited statistically significant alterations in cell migration, with diminished migration observed in the EV-treated group between 10 and 24 h. Statistical analysis was performed using the two-way ANOVA test with a post-test by Šídák, both with a significance level of *p* < 0.05. Significance levels obtained in the test were * *p* = 0.0125; ** *p* = 0.0013; *** *p* = 0.0002/*p* = 0.0004; **** *p* < 0.0001.

In the cell migration and invasion assays, noticeable changes were observed in PTC cell lines. After 120 h of pre-treatment with hIDPSC-EVs, the BCPAP cell line displayed a reduced migration capacity compared to the control group. However, this change was transient, occurring only between 10 and 24 h of analysis, with the behavior returning to a pattern similar to that of the control group at other assessed intervals (0 to 10 h and from 24 to 32 h). In contrast, the migration capacity of the remaining groups was not significantly different from the control group (Figure 5). 

**Figure 6:**
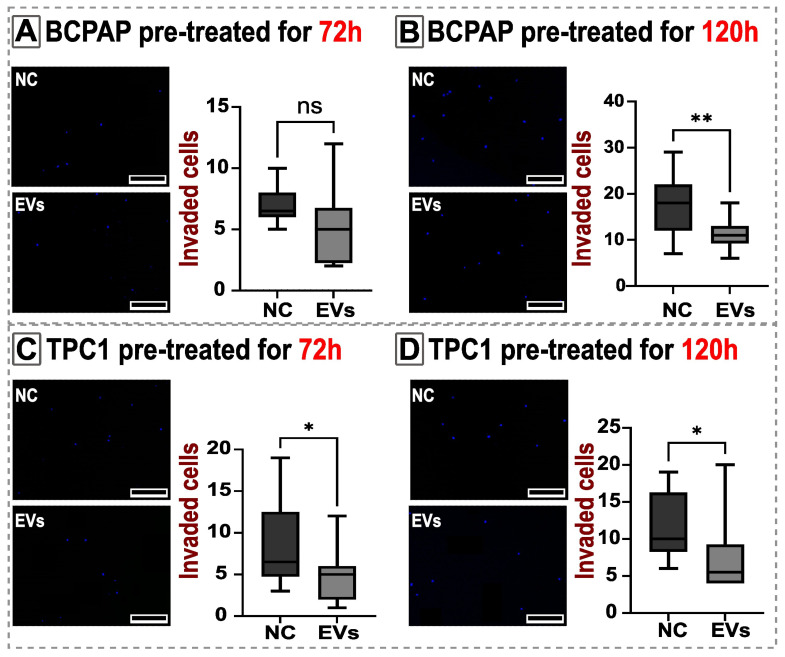
Invasion by BCPAP and TPC1 cells. The invasion assay showed a reduction of invaded cells after EV treatment in almost all treatments (**B**–**D**). Only BCPAP pre-treated for 72 h did not show a significant alteration in invasion capacity (**A**). The left panels show representative images of nuclei in blue (DAPI) of invaded cells of the negative control (NC) and EV-treated cells (EVs). Invaded cells were defined as those that moved through the Matrigel coat and the Transwell pore (8 μm) to the lower side of the Transwell membrane. In the left panels, black bars represent a scale bar of 500 μm inserted using ImageJ/FIJI software. The whiskers in the boxplot represent minimum and maximum values. Statistical analysis was performed using one-way ANOVA with a post hoc Tukey’s test, both with a significance level of *p* < 0.05. Significance levels obtained in the test were ns *p* > 0.05; * *p* = 0.0210, *p* = 0.0292; ** *p* = 0.0018.

In the cell invasion assay, only the BCPAP cell line pre-treated for 72 h showed no statistical difference between the treated and control groups. Conversely, all other groups exhibited a statistically significant decrease in invaded cells after EV treatment (Figure 6).

## 3. Discussion

Human IDPSC-EVs are a promising and underexplored tool in cancer research. In this study, we investigated the effects of hIDPSC-EVs on two well-documented in vitro PTC models: (1) the BCPAP cell line, which harbors the *BRAF^V600E^* mutation, found in 40–60% of PTC cases [27,28], and (2) the TPC1 cell line, which features the *RET/PTC1* rearrangement, present in approximately 20% of PTC cases [28,29]. Both driver mutations (*BRAF^V600E^* and *RET/PTC*) lead to activation of the MAPK pathway, driving cell proliferation and tumorigenic behavior [27,28,29].

Initially, we analyzed the particle size profiles of hIDPSC-EVs. The medium particle size of our hIDPSC-EV was 210.2 nm ± 5.6 nm and the median of the particle size was 202.7 ± 5/8 nm (Supplementary Figure S2), which, according to size classification, would fit with the operational term ‘Large-EV’ (>200 nm), although median size standard deviation encompasses ‘Small-EVs’ (<200 nm) [14]. 

After size characterization, we evaluated the capacity of PTC cells to internalize (uptake) hIDPSC-EVs. In this assay, we showed that both PTC cell lines internalized hIDPSC-EVs in a time-dependent manner. The BCPAP cell line showed more efficient internalization of hIDPSC-EVs followed by more pronounced gene expression regulation (in the qPCR analysis) compared to TCP1 cells. Because we did not investigate the specific mechanisms of hDPSC-EV internalization by PTC cell lines in this study, we were not able to explain whether the uptake ratio differs due to the specific features of each cell line studied. Cells can internalize EVs through various mechanisms, including phagocytosis, clathrin- and caveolin-mediated endocytosis, interactions with lipid rafts, and macropinocytosis [30]. They can also deliver cargo by fusing with the plasma or endosomal membrane, triggering a phenotypic response [30]. Considering the observed differences between the two cell lines, it is crucial to investigate the mechanisms of EV internalization in future studies as this may have crucial implications for future therapeutic applications. 

Following the confirmation of uptake, we investigated the transcriptional alterations following hIDPSC-EV treatment using two qPCR-based arrays. In addition to the modulation of several genes in these arrays, our gene enrichment analysis revealed the modulation of only two pathways (WebGestalt names: the Wnt pathway and cellular responses to stimuli). The Wnt pathway, positively modulated in BCPAP cells after 72 h of treatment, plays a pivotal role in adult tissue homeostasis by regulating processes, such as cell proliferation, polarity, stem cell activation, and self-renewal [31,32,33,34]. The canonical Wnt pathway initiates with Wnt proteins binding to the Frizzled receptor (Fzd) and its coreceptor LRP5/6 (LDL-Receptor-related protein) [31,32,33,34]. This binding event triggers phosphorylation of the Dishevelled (Dvl) protein, recruiting Axin and leading to disassembly of the degradation complex (composed of APC, Axin, CK1, and GSK3B) [31,32,33,34]. In the absence of this complex, β-catenin accumulates in the cytoplasm, translocates to the nucleus, and forms a complex with T-cell factor (TCF) and lymphoid enhancer factor (LEF), thereby activating Wnt target genes, including the proto-oncogene *MYC* [31,32,33,34]. In BCPAP cells treated for 72 h, genes from the canonical Wnt pathway (*FZD1*, *DVL1*, *AKT2*, *CTNNB1*, *LEF1*, *MYC*) and the non-canonical Wnt pathway (*RHOA*, related to cytoskeletal dynamics and increased migration and invasion) were upregulated, suggesting pro-tumor behavior [31,32,33,34]. However, assays conducted on BCPAP cells treated with EVs for 72 h did not confirm an increase in proliferation, migration, or invasion. Additionally, upregulation of APC and GSK3β, components of the degradation complex, was observed, possibly indicating a homeostatic mechanism [32,35]. The absence of proteomic analysis limits our understanding of whether the upregulated genes were translated into proteins and active at the evaluated time points. Interestingly, no significant differences were observed in the subsequent 120 h analysis, indicating a recovery of transcriptional signaling over time. 

Other transcriptional alterations were observed in *TPC1* cells treated with hIDPSC-EVs for 120 h, and gene enrichment indicated downregulation of *CDKN2A* and *TP53* genes, which are critical for tumor suppression. *TP53* encodes p53, and *CDKN2A* encodes p14ARF and p16INK4A, which are proteins essential for apoptosis, cell cycle arrest, and senescence [36,37,38]. The downregulation of these genes is expected to negatively affect cell proliferation. However, cell cycle and proliferation assays have not confirmed this predicted behavior [36,37,38]. Another gene downregulated in the enriched pathway for TPC1 at 120 h was vascular endothelial growth factor (*VEGFA*). When upregulated, VEGF is a key mediator of angiogenesis and directly correlates with tumor progression (including metastasis). In contrast to our observations, the downregulation of p16INK4A and p53 is usually related to the upregulation of *VEGFA* [39,40]. Besides that, few conclusions can be drawn after this, as the expression of *VEGFA* is related to a more complex mechanism dependent on other cellular types and their dynamics with cancer cells [41]. 

Interestingly, we observed positive results for cell motility and invasive behavior in both PTC cell lines. The Transwell Matrigel assay showed a decreased number of invading cells, indicating that hIDPSC-EV treatment reduced the ability of PTC cell lines to degrade the extracellular matrix (ECM) and migrate to other tissues [42,43]. A reduction in cell invasion may directly impact the crucial metastatic cascade that involves cell penetration into the ECM and invasion of surrounding tissues [42,44,45], demanding several microenvironmental alterations, such as changes in adhesion proteins, cytoskeleton reorganization, epithelial–mesenchymal transition (EMT), and proteolytic enzyme production [44]. Similar to our findings, other studies exploring the anticancer potential of dental pulp stem cell (DPSC) lysate and conditioned media have shown a decrease in cell invasion and migration, along with decreased cell proliferation and increased pro-apoptotic activity in in vitro models of lung and colorectal cancer [46,47].

Overall, our study showed that hIDPSC-EVs decreased the invasive capacity of PTC cell lines without enhancing their proliferation or other pro-tumor behaviors. These results suggest that hIDPSC-EVs have significant potential for cancer treatment, especially because of the successful internalization of hIDPSC-EVs by PTC cell lines, indicating that these EVs could be further explored as nano-delivery tools for chemotherapeutic molecules in the future. Besides this in vitro evidence, in vivo studies should be carried out to unveil the general safety of hIDPSC-EVs, their delivery, and their role in in vivo models of PTC. 

## 4. Materials and Methods

### 4.1. Cell Culture 

For this study, two thyroid carcinoma cell lines were used: BCPAP “https://www.cellosaurus.org/CVCL_0153” (accessed on 20 March 2024), originating from a poorly differentiated thyroid carcinoma (PDTC), and TPC1 “https://www.cellosaurus.org/CVCL_6298” (accessed on 20 March 2024), derived from a typical papillary thyroid carcinoma (PTC). Both cell lines were cultured in 75 cm^2^ flasks (Corning^®^, Corning, NY, USA, catalog no. 430641U) in RPMI Medium 1640 (Gibco^TM^, Grand Island, NY, USA, catalog no. 22400071) supplemented with 10% fetal bovine serum (FBS, Gibco^TM^, catalog no. 12657029) and 1% penicillin–streptomycin Gibco^TM^, Grand Island, NY, USA, catalog no. 15140122). Once 80% confluence was reached, cells were washed with PBS, catalog no. 10010023) and detached using TrypLE™ Select Enzyme (Gibco^TM^, Grand Island, NY, USA, catalog no. 12563011). 

### 4.2. Human IDPSC Culture and Characterization and Extracellular Vesicle Isolation

Human IDPSCs were isolated from the deciduous teeth of human dental pulp using the method described by Kerkis et al. (2007) [48]. Next, cells were cultivated until the fifth passage (P5) in complete culture medium (DMEM/F-12 culture medium, supplemented (Gibco^TM^, Grand Island, NY, USA, catalog no: 11320082) with 10% fetal bovine serum (FBS, Corning^®^, Corning, NY, USA catalog no: 35-096-CV), and 1% of penicillin–streptomycin solution. The multipotency of hDPSCs was evaluated based on their ability to differentiate into osteogenic and chondrogenic lineages, as described by Ciuffreda et al. (2016) [49], with minor modification. hDPSCs were also characterized based on the expression of their surface markers, as described by Dominici et al., 2006 [50]. Flow cytometry was used to assess positive (CD105, CD73, and CD90) and negative (CD45, CD34, CD11b, CD19, and HLA-DR) markers. Data acquisition was performed using a BD FACSCalibur flow cytometer (BD Biosciences, San Jose, CA, USA). Characterization results are available in Supplementary Figure S8. 

For the conditioned medium obtention, after reaching 80% confluence, the complete culture medium from hIDPSC was replaced with fresh basal culture medium without FBS (DMEM/F-12 culture medium supplemented with 1% penicillin–streptomycin) after three washes with PBS. After 24 h, the conditioned culture medium (CCM) was collected and filtered using filters with a 0.22 μm pore size (Millipore, Burlington, MA, USA, catalog no. S2GPU02RE). CCM was subjected to hIDPSC-EV isolation by using an adaptation of the method described by Koga et al. [51]. EV was isolated using a Himac CP80WX ultracentrifuge (Eppendorf Himac, Hitachinaka, Japan) and ultracentrifuged for 1 h at 100,000× *g* (RCF) at 4 °C. The hIDPSC-EV suspension was then washed with PBS and ultracentrifuged again following the same protocol. Finally, the EV solution was filtered using PVDF filters with a 0.22 μm pore size and stored in 100 µL aliquots at −80 °C.

### 4.3. EV Quantification and Physical Characterization through NTA

The hIDPSC-EV size and particle concentration were assessed using NanoSight NS300 (Malvern Panalytical, Malvern, Worcestershire, UK), a widely used tool powered by the Nanoparticle Tracking Analysis (NTA) method. For the analysis, EVs were diluted to a 1:20 dilution ratio using deionized water. The focus and capture settings were adjusted using NanoSight NTA software v3.3 (Malvern Panalytical, Malvern, Worcestershire, UK), and five videos of 60 s were captured for the software analysis. Additionally, the relative concentration of hIDPSC-EV protein mass was determined using the Bicinchoninic Acid (BCA) Protein Assay Kit (Thermo Scientific™, Waltham, MA, USA, catalog no. 23227). Human IDPSC-EVs’ total protein was previously extracted using RIPA buffer (Thermo Scientific™, Waltham, MA, USA, catalog no. 89900) following the manufacturer’s instructions. Analysis was performed using an Infinite 200 PRO^®^ plate reader at a wavelength of 562 nm (Tecan, Männedorf, Switzerland, catalog no. 30050303). The transmission electron microscopy (TEM) of hIDPSC-EVs was performed following the protocol described by Jung et al. (2018) [52].

### 4.4. EV Labeling

For the uptake assay, hIDPSC-EVs were labeled with the Vybrant™ DiO dye solution (Invitrogen™, Waltham, MA, USA, catalog no. V22886). For this, 1 μL of the Vybrant™ DiO dye solution was gently mixed with 200 μL of isolated EVs and incubated for 20 min at 37 °C. To stop the labeling reaction, the suspension of EVs was incubated for one minute with 1 mL of 1% BSA in PBS. To remove the excess of unlabeled dye, EVs were washed in 11.8 mL of PBS and ultracentrifuged for 1 h at 100,000× *g* (RCF) at 4 °C, thus discarding the supernatant (about 11.9 mL). EVs were stored in small aliquots at −80 °C for further use. 

### 4.5. Uptake Assay

To verify the uptake of hIDPSC-EVs by PTC cells, BCPAP and TPC1 were incubated with 50 μg/mL EVs-DiO and subjected to confocal microscopy, time-lapse confocal microscopy, and flow cytometry.

#### 4.5.1. Confocal Microscopy

Regular confocal microscopy analysis was performed to visualize 2D and 3D EVs within the cell cytoplasm. For this, 10,000 PTC cells (BCPAP and TPC) were seeded per well in a Nunc™ Lab-Tek™ Chamber Slide 8-well configuration (Thermo Scientific™, Waltham, MA, USA, catalog no. 177380) containing 300 μL of complete culture medium. After cell adhesion, the culture medium was replaced with 300 μL of complete culture medium containing 50 μg/mL EVs-DiO. After 24 h of treatment, EVs-DiO solution was removed, and the cells were washed with PBS to remove residual EVs-DiO. Cells were fixated in a 4% paraformaldehyde (PFA) solution for 15 min. The fixed cells were washed twice with PBS to remove PFA and stained with a Phalloidin Alexa Fluor 647 solution (Thermo Scientific™, Waltham, MA, USA, catalog no. A22287) prepared as follows: 0.5 µL of the Phalloidin 400× stock solution, previously dissolved in 150 µL of DMSO, in 200 µL of PBS. The cells were incubated with the solution of Phalloidin in PBS for 1 h at room temperature (20–25 °C), washed twice with PBS, counterstained with 1 µg/mL of Hoechst 33342 (Thermo Scientific™, Waltham, MA, USA, catalog no. H1399) solution for 5 min, and washed three times with PBS to remove excess Hoechst 33342. Finally, the well structure of the chamber was detached from the slide and transferred to a coverslip containing 10–20 µL of ProLong™ Glass Antifade Mountant (Thermo Scientific™, Waltham, MA, USA, catalog no. P36980). Slides were kept at 4 °C until the analyses. The 2D, 3D, and orthogonal views were captured using a Leica TCS SP8 Laser Scan Confocal Microscope (Leica Microsystems, Wetzlar, Germany), with a flat apochromatic 63×/1.20 water immersion objective. Fluorescence and image configuration settings were made using Leica LAS X software v. 5.2.2 (Leica Microsystems, Wetzlar, Germany).

#### 4.5.2. Time-Lapse Confocal Microscopy

PTC cells (BCPAP and TPC1) were seeded in triplicate on Black Nunc MicroWell 96-Well Optical-Bottom Plates (Thermo Scientific™, Waltham, MA, USA, catalog no. 165305) at a density of 3000 cells/well. After cell adhesion, the culture medium was replaced with a fresh culture medium containing 50 µg/mL of EVs-DiO. The plate was immediately placed into the CO_2_ compartment of a confocal microscope TCS SP8 (Leica Microsystems, Wetzlar, Germany). The time-lapse settings were adjusted using the Leica LAS X software v. 5.2.2 (Leica Microsystems, Wetzlar, Germany), and images of each well were captured every 45 min in several Z-axis slices for 48 h.

LAS X software was employed to convert the 3D images from each time point into 2D images. This was achieved by selecting slices that exhibited internal fluorescence based on the data from the final time point (48 h). Subsequently, these 2D images were analyzed using a ImageJ macro (ImageJ/FIJI v.1.54f) to calculate the mean fluorescence intensity (MFI) of EVs-DiO and Control-DiO using a code developed for this purpose https://github.com/MichelliRT/EVsUptakeTimeLapseConfocal/tree/main (accessed on 23 May 2024). 

### 4.6. Transcriptional Effects Promoted the EVs

#### 4.6.1. RNA Extraction and Quality Control

For RNA extraction, cells were cultured in 25 cm^2^ flasks and treated with EVs as described previously. After 72 or 120 h of treatment, the medium was removed and the cells were washed twice with warm PBS. Next, 1 mL of TRIzol™ Reagent (Invitrogen™, Waltham, MA, USA, catalog no. 15596026) was added to the cells. The cell lysate was transferred to DNase/RNase-free microtubes and mixed with 0.2 mL of chloroform through inversion for 10 min, followed by centrifugation at 10,000× *g* for 15 min at 4 °C. The aqueous phase was transferred to a new microtube, mixed through inversion with isopropanol (0.5 mL) for 10 min at 4 °C, centrifuged at 10,000× *g* for 30 min at 4 °C, and the supernatant was discarded. The RNA pellet was washed three times with 1 mL of cold 90% ethanol and centrifuged for 10 min as previously described. After the final wash, the RNA pellet was air-dried at room temperature and resuspended in 50 µL of DEPC-treated water. RNA concentration was assessed using 1 µL of the sample on a NanoDrop Oneᶜ Microvolume UV-Vis Spectrophotometer (Thermo Scientific™, Waltham, MA, USA). The remaining RNA was stored at −80 °C.

#### 4.6.2. cDNA Synthesis

After quantification, 500 ng of RNA was used to synthesize cDNA using the SuperScript™ III First-Strand Synthesis System (Invitrogen, catalog no. 18080051), according to the manufacturer’s instructions. To ensure the effectiveness of cDNA synthesis, real-time PCR (qPCR) was performed using SYBR™ Green reagent (Invitrogen) and 500 nM of each of the following GAPDH gene segments: forward primer 5′-GAGTCCACTGGCGTCTTCAC-3′ (Invitrogen) and reverse primer 5′-GCACTGTGGTCATGAGTCCTTC-3′ (Invitrogen). qPCR reaction was performed using the QuantStudio™ 3 Real-Time PCR System (Applied Biosystems™, Waltham, MA, USA, catalog no. A28567) with the following temperature ramp: (1) 50 °C for 1 min, (2) 95 °C for 2 min, and (3) 40 cycles of 95 °C for 15 s followed by 60 °C for 1 min.

#### 4.6.3. Molecular Profiling through qPCR Arrays

To evaluate the eventual transcriptional changes in PTC cell lines promoted by the treatment with EVs at two different incubation times (72 and 120 h), two distinct qPCR arrays were used, comprising (1) 92 genes involved in molecular mechanisms of cancer (TaqMan™ Array Human Molecular Mechanisms of Cancer, Applied Biosystems™, catalog no. 4414161) and (2) 92 genes involved in metastasis-related mechanisms (TaqMan™ Array Human Tumor Metastasis, Applied Biosystems™, catalog no. 4414098). Each array also included four endogenous candidate control genes (18s, *GAPDH*, *HPRT1*, and *GUSB*). The plates were prepared according to the manufacturer’s instructions, using 1 ng of cDNA (estimated based on the concentration of RNA used for cDNA synthesis) and 10 μL of TaqMan^®^ Gene Expression Master Mix (Applied Biosystems, catalog no. 4369016) were used per well. Control (untreated) and EV-treated plates were prepared and analyzed for each cell line and time point using the same reagents and qPCR cycle conditions on a QuantStudio™ 3 Real-Time PCR System. Cycle threshold (Ct) values were obtained using the Applied Biosystems 7500 Real-Time PCR System software v1.1 (Thermo Scientific™, Waltham, MA, USA) and used to determine the log2fold change in each target gene, calculated in Excel (Microsoft, Redmond, WA, USA). Genes were normalized to the Ct values of the endogenous genes *GUSB* and *HPRT1*. 18s and *GAPDH* were not used in the analysis. All valid values (valid amplification status) of the log2fold change were used to obtain heatmap. Heatmaps were generated using RStudio v2023.06.2 with R v4.3.3 and the pheatmap function from the ComplexHeatmap package v2.18.0. Hierarchical clustering was performed by using the complete linkage method. Pathway enrichment analysis was performed using the WebGestalt (WEB-based GEne SeT AnaLysis Toolkit) platform, employing the Gene Set Enrichment Analysis (GSEA) method to identify regulated pathways. The genes of the enriched pathways are presented in graphs with the value of their log2FC, generated using GraphPad Prism software version 10.0.0 (GraphPad Software). 

### 4.7. Cell Viability Analysis

Cells were cultured in 6-well plates at seeding densities of 60,000 cells/well for 72 h treatments and 20,000 cells/well for 120 h treatments to accommodate their rapid proliferation rates and treatment durations. The cells were treated with 50 μg/mL EVs., collected using TrypLE™ reagent, and stained using the LIVE/DEAD™ Fixable Aqua Dead Cell Stain Kit (Invitrogen, catalog no. L34957) according to the manufacturer’s instructions. Flow cytometry was performed using a BD FACSCanto™ II (BD Biosciences, San Jose, CA, USA) at 367/526 nm. Fluorescence calibration involved a dead cell control (heat-shocked PTC cell lines) and an unstained control. For each sample, 10,000 events inside of a gate were traced using FSC/SSC. FlowJo software V10 was used for data analysis, employing dead cell controls to establish live/dead gates and calculate viable cell percentages. The gate strategies are shown in Supplementary Figure S9. 

### 4.8. Cell Proliferation 

To evaluate the proliferative profile of PTC cell lines after EV treatment, two different methods were used to determine cell proliferation: CellTrace^TM^ Violet, a colorimetric assay that permanently labels cells with fluorescent stains to track generations or divisions in vivo, and the Cell Counting Kit-8 (CCK-8), a colorimetric assay that determines the number of viable cells in cell proliferation assays.

#### 4.8.1. CellTrace^TM^ Violet Assay

For the CellTrace^TM^ Violet assay (Invitrogen™, catalog no. C34557), the cells were harvested and 100,000 cells were separated from the pool of cells for the unmarked control. The remaining cells were stained with CellTrace Violet reagent (1 μL of dye for 1 × 10^6^ cells), according to the manufacturer’s instructions. A small number of cells were separated to confirm the labeling efficiency. After the confirmation of cell labeling, 100,000 cells were fixed with 4% PFA within 15 min (T0 sample). The remaining fresh and labeled cells were seeded in triplicate for the time points of 48, 72, 96, and 120 h for both treatments (control and 50 μg/mL EVs). After cell adherence (~6 h), the culture medium was changed to a serum-free culture medium before the treatments to synchronize the cell cycle. At each time point, cells were harvested, fixated with PFA 4%, and stored in PBS at 4 °C. At the end of the experiment, the cells were analyzed using a BD FACSCanto™ II (BD Biosciences, San Jose, CA, USA) at 405/450 nm. For each sample, 10,000 events were acquired inside of a predefined gate and traced using the FSC/SSC. Data analysis using FlowJo software V10 provided the median fluorescence intensity (MFI) for each replicate (the gate strategy is provided in Supplementary Figure S9). MFI statistical analysis was performed using GraphPad Prism software v10.0.0 (GraphPad Software). 

#### 4.8.2. Cell Counting 8 Assay

For the CCK-8 assay, a calibration curve was obtained for both PTC lineages, correlating the absorbance values (obtained by the CCK-8 assay) with the number of seeded cells. After determining the curve, the cells were seeded in 96-well plates at a 450 cells/well concentration. The cells were treated for 24, 48, 72, 96, and 120 h with 50 μg/mL of EV in triplicate. At each time point, the culture medium was discarded, and a fresh culture medium containing 10% CCK-8 reagent (Sigma-Aldrich^®^, St. Louis, MO, USA, catalog no. 96992) was added to the cells. After 4 h, absorbance was measured at 450 nm using an Infinite 200 PRO^®^ plate reader (Tecan, Männedorf, Switzerland, catalog no. 30050303). Data analyses were performed by applying the obtained absorbance values to a fourth-grade polynomial equation using a pre-established calibration curve.

### 4.9. Cell Cycle 

To assess the impact of EV treatment on the cell cycle, PTC cell lines were cultured in 6-well plates and subjected to a 24 h starvation period before EV treatment (outlined in the Cell Viability Analysis section). The cells were fixed in cold 70% ethanol for 1 h, washed with PBS, and stained with 0.5 mL of FxCycle™ PI/RNase Staining Solution (Invitrogen, catalog no. F10797) for 30 min. Subsequently, the stained cells were analyzed using the BD FACSCanto™ II flow cytometer (BD Biosciences, San Jose, CA, USA), recording 10,000 events per sample at 488 nm excitation and 585 nm emission. Data analysis was conducted using the cell cycle analysis tool of the FlowJo V10 software to calculate the percentage of cells in the G0/G1, S, and M/G2 phases. The percentages were subjected to statistical analysis using GraphPad Prism version 10.0.0 (GraphPad Software). The analysis was performed in triplicate. 

### 4.10. Mitochondrial Membrane Potential and ROS Generation Analyses CM-H2DCFDA

To investigate whether hIDPSC-EV treatment promoted changes in energy cell metabolism, mitochondrial membrane potential (*ΔΨm*) and reactive oxygen species (ROS) generation were analyzed. For both assays, 30,000 cells/well (BCPAP or TPC1) were seeded in 6-well plates. The cells were treated for 72 h with 50 µg/mL of EVs. Cells were then incubated for 20 min with the MitoTracker^®^ Green FM (Invitrogen™, catalog no. M7514) to assess the *ΔΨm* or 30 min with 5 µg of the general oxidative stress indicator CM-H2DCFDA to assess ROS generation (Invitrogen™, catalog no. C6827). After the incubations, cells were harvested, washed in PBS, and analyzed in flow cytometer BD FACSCanto™ II (BD Biosciences, San Jose, CA, USA) at 490/516 nm, with 10,000 events analyzed per analysis. 

### 4.11. Wound Healing Assay

To verify whether EVs can affect the migration ability of PTC cell lines, BCPAP and TPC1 cells were subjected to the wound healing assay. For this purpose, 180,000 cells were seeded per well in a 24-well plate. At a confluence of nearly 90%, a scratch was made in each well using a 200 μL pipette tip. The wells were washed thrice with warm PBS to completely remove the detached cells. Next, the cells were cultured in RPMI medium with 2% FBS and immediately placed into the CO_2_ compartment of a confocal microscope TCS SP8 (Leica Microsystems, Wetzlar, Germany). The time-lapse settings were adjusted using the Leica LAS X software v. 5.2.2 (Leica Microsystems, Wetzlar, Germany), and images of four wound spots from each well were taken every 30 min for 48 h. Wound area closure was evaluated using Image J/FIJI by applying the macro described by Pijuan et al. [43]. The experimental design included (1) EV-primed cells, BCPAP or TPC1 pre-treated for 72 and 120 h with 50 µg/mL of EVs and (2) a negative control, cells not treated with EVs. 

### 4.12. Transwell Invasion Assay

To verify whether hIDPSC-EVs can affect the invasion ability of PTC cell lines, BCPAP and TPC1 cells were subjected to the Transwell invasion assay, as described by Pijuan et al. [43]. Initially, the upper chamber of an 8.0 μm Transwell insert Corning^®^, Corning, NY, USA, catalog no. CLS3428) was coated with 100 μL of Corning^®^ Matrigel^®^ Matrix (final concentration of 300 μg/mL, Corning^®^, Corning, NY, USA, catalog no. 354234) and allowed to gel overnight at 37 °C. Subsequently, 40,000 cells (primed and control groups) were seeded on top of the gel matrix in 300 μL of serum-free medium. The lower chamber received 800 μL of RPMI medium supplemented with 10% FBS, and the setup was incubated for 24 h. Non-invading cells on the upper surface of the insert were removed using a cotton swab, and the insert was washed with PBS. Cells that invaded the lower surface were fixed for 15 min with 4% PFA, stained with 1 μg/mL of DAPI (Sigma-Aldrich^®^, D9542), and imaged using a fluorescence microscope (Nikon, Tokyo, Japan). The number of invading cells was determined using ImageJ/FIJI software (ImageJ/FIJI v.1.54f), following the protocol described by Pijuan et al. [43].

### 4.13. Statistical Analysis

Statistical analyses of proliferation, cell cycle, viability, MitoTracker (ΔΨm), and ROS (CM-H2DCFDA) were performed using the Mann―Whitney test with a significance level of *p* < 0.05 using GraphPad Prism. The migration assay was analyzed using the two-way analysis of variance (ANOVA) with Tukey’s post hoc test (*p* < 0.05) using GraphPad Prism version 10.0.0 software. The cell invasion assay was analyzed using one-way ANOVA with Šídák’s post hoc test (*p* < 0.05). All statistical analyses were conducted in triplicate (*n* = 3). Gene expression analysis was conducted using the WebGestalt Webservice “https://www.webgestalt.org/” (accessed on 20 July 2024).

## Data Availability

The original contributions presented in the study are included in the article/Supplementary Material, further inquiries can be directed to the corresponding author/s.

## Supplementary Materials

The supporting information can be downloaded at: https://www.mdpi.com/article/10.3390/ijms25158178/s1.
