# Supplementary material for: Exploring the Therapeutic Potential of Extracellular Vesicles Derived from Human Immature Dental Pulp Cells on Papillary Thyroid Cancer
Source: Int J Mol Sci. 2024 Jul 26;25(15):8178. doi: 10.3390/ijms25158178 (PMC11311836; doi:10.3390/ijms25158178)
Supplement: Supplementary file 1 [file ijms-25-08178-s001.zip › ijms-3103661-Supplementary Materials.pdf]

Supplementary data: Exploring the therapeutic potential of Extracellular Vesicles Derived From Human Immature Dental Pulp Stem Cells on Papillary Thyroid Cancer

NANOSIGHT

Rodada4-010823 2023-08-01 14-47-44 2023-08-01 17-51-29 2023-08-01 18-19-41

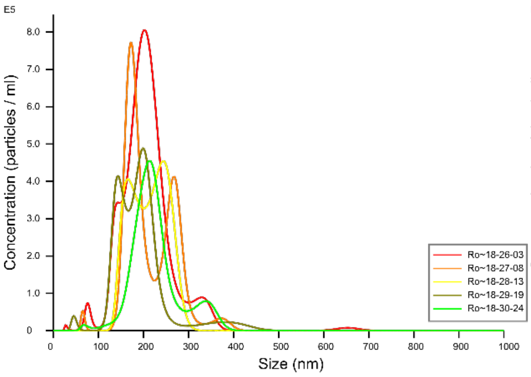

FTLA Concentration / Size graph for Experiment: Rodada4-010823 2023-08-01 14-47-44 2023-08-01 17-51-29 2023-08-01 18-19-41

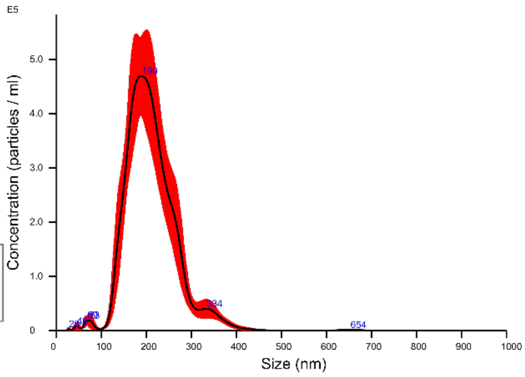

Averaged FTLA Concentration / Size for Experiment: Rodada4-010823 2023-08-01 14-47-44 2023-08-01 17-51-29 2023-08-01 18-19-41
Error bars indicate + / -1 standard error of the mean

Included Files

Rodada4-010823 2023-08-01 14-47-44 2023-08-01 17-51-29 2023-08-01 18-26-03
Rodada4-010823 2023-08-01 14-47-44 2023-08-01 17-51-29 2023-08-01 18-27-08
Rodada4-010823 2023-08-01 14-47-44 2023-08-01 17-51-29 2023-08-01 18-28-13
Limited file list. Full list printed on Page 2.

Details

NTA Version:	NTA 3.4 Build 3.4.4
Script Used:	Rodada 4_010823.txt
Time Captured:	18:19:41 01/08/2023
Operator:	and
Pre-treatment:	
Sample Name:	rodada4_010823_ 15 graus
Diluent:	
Remarks:	

Capture Settings

Camera Type:	sCMOS
Laser Type:	Blue488
Camera Level:	11
Slider Shutter:	890
Slider Gain:	146
FPS	25.0
Number of Frames:	1498
Temperature:	15.0 - 15.3 °C
Viscosity:	(Water) 1.128 - 1.135 cP
Dilution factor:	Dilution not recorded
Syringe Pump Speed:	50

Analysis Settings

Detect Threshold:	40
Blur Size:	Auto
Max Jump Distance:	Auto: 11.9 - 14.2 pix

Results

Stats: Merged Data

Mean:	209.7 nm
Mode:	189.1 nm
SD:	56.9 nm
D10:	149.9 nm
D50:	202.4 nm
D90:	275.4 nm

Stats: Mean +/- Standard Error

Mean:	210.2 +/- 5.6 nm
Mode:	206.1 +/- 11.6 nm
SD:	54.9 +/- 3.8 nm
D10:	153.1 +/- 6.3 nm
D50:	202.7 +/- 5.8 nm
D90:	274.7 +/- 12.4 nm
Concentration:	5.51e+07 +/- 6.79e+06 particles/ml
	3.0 +/- 0.4 particles/frame
	3.7 +/- 0.5 centres/frame

Figure S1. Determination of extracellular vesicle diameter and concentration using NanoSight NS300.

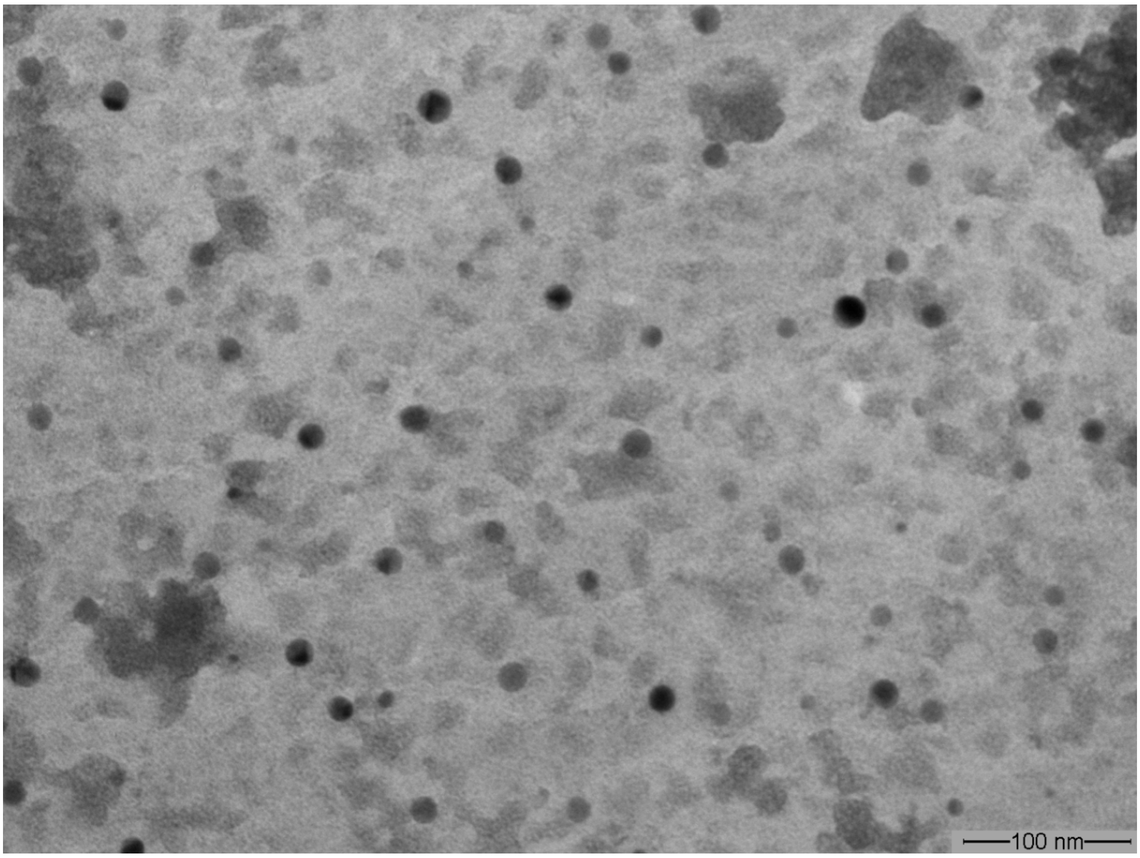

Figure S2. Transmission electron microscopy (TEM) of hIDPSC-EVs.

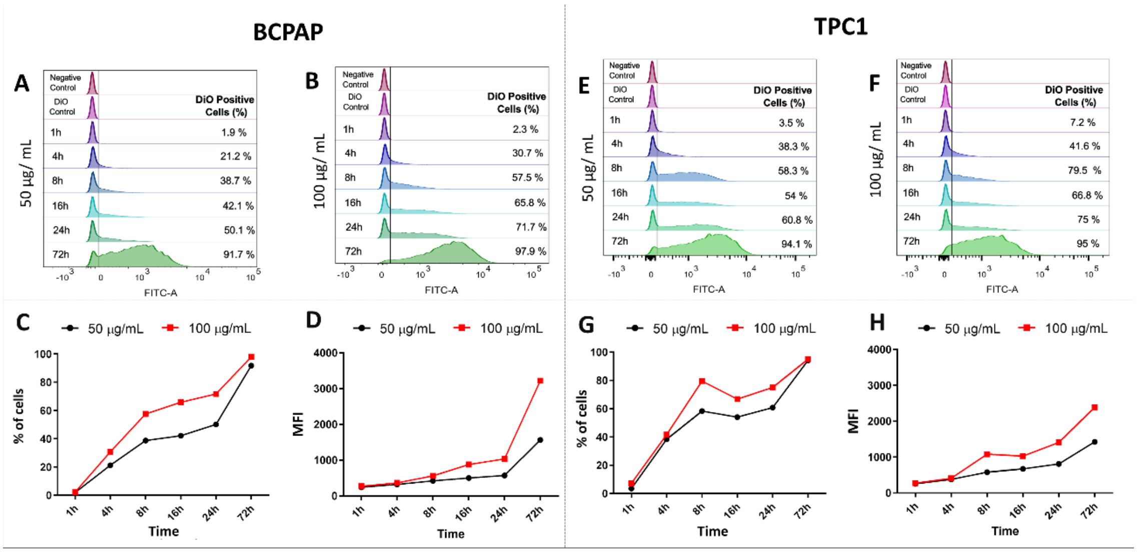

Figure S3. Extracellular vesicle uptake. EVs-DiO internalization was evaluated by flow cytometry using two concentrations of EV-DiO (50 and 100 µg/mL) at various time points (1, 4, 8, 16, 24, and 72 h). This assay assessed the concentration- and time-dependent internalization of EVs-DiO by BCPAP and TPC1 cells. In the histograms represented in panels A, B, E, and F, an increase in the number of DiO-positive cells over time was evident. Additionally, the percentage of DiO-positive cells was higher at 100 µg/mL than at 50 µg/mL, indicating the concentration-dependent internalization of EVs. Using the median fluorescence intensity (MFI) obtained from the FITC channel of FACS Canto II

and the percentage of cells positively labeled with EV-DiO, graphs C, D, G, and H were generated. These graphs show that, although the pattern of internalization over time was similar, both the percentage of DiO-positive cells and MFI were higher with a greater concentration of EV-DiO in the BCPAP cell line. The two parameters analyzed, the percentage of DiO-positive cells and MFI, were designed to answer the following key questions: whether all cells internalized EVs-DiO, the timing of this process, whether EV internalization continued after the initial uptake, and at what point it reached a plateau. Despite these interesting results, the analysis was performed in uniplicate, preventing statistical analysis and proper correlation between the qualitative observations.

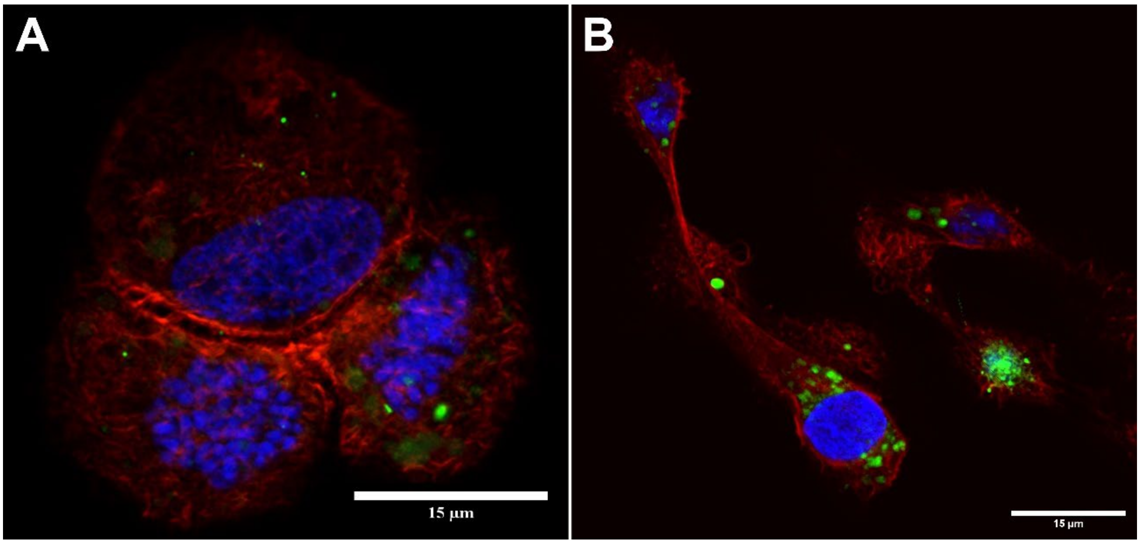

Figure S4. Super-resolution microscopy images were captured using a Zeiss LSM880 Airyscan Inverted Microscope with a 63x objective. These figures illustrate the internalization of EVs in TPC1 cells. EVs are shown in green (EVs-DiO), the cytoskeleton is stained red with Phalloidin Alexa Fluor 647, and the nucleus is stained blue with Hoechst 33342.

TIME-LAPSE REPRESENTATIVE IMAGES

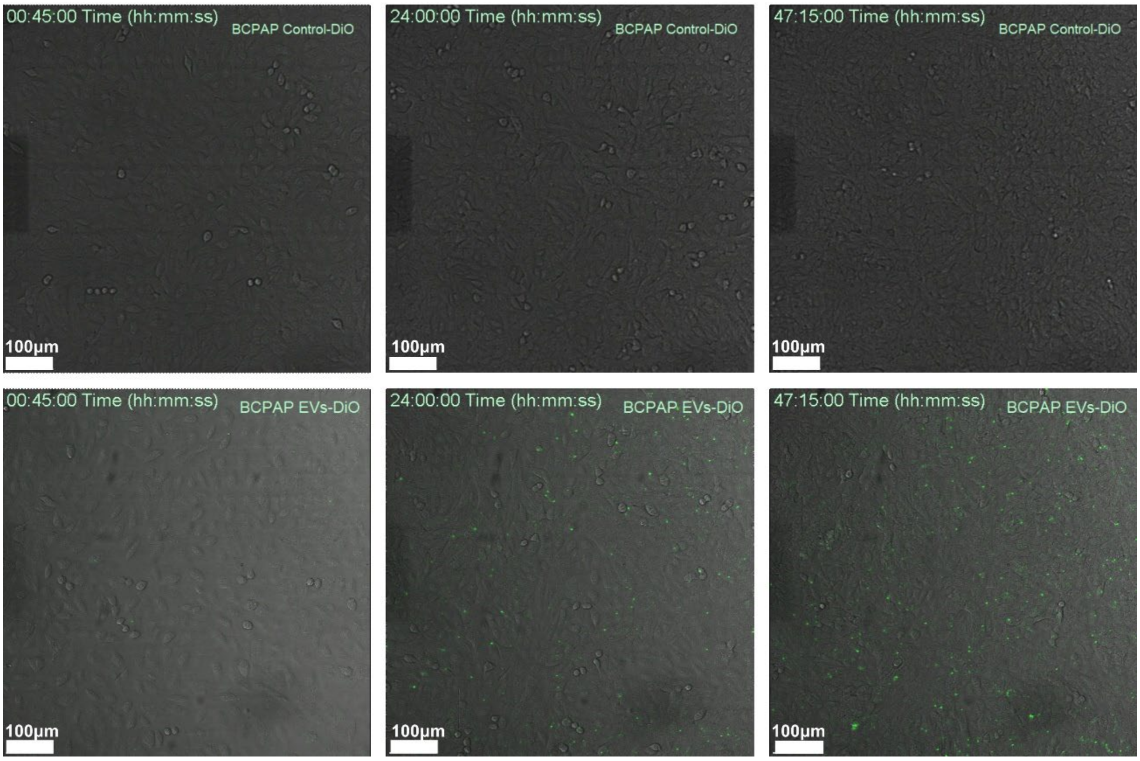

Figure S5. Time-lapse representative images. The images show Control-DiO uptake at 45 min, 24 h, and 47 h 15 min in the first row, and EV-DiO uptake at the same time points in the second row. Control-DiO did not exhibit fluorescent signals, whereas EV-DiO did, indicating that EV-DiO was internalized, and the fluorescence signals observed were from EVs and not from the dye artifacts.

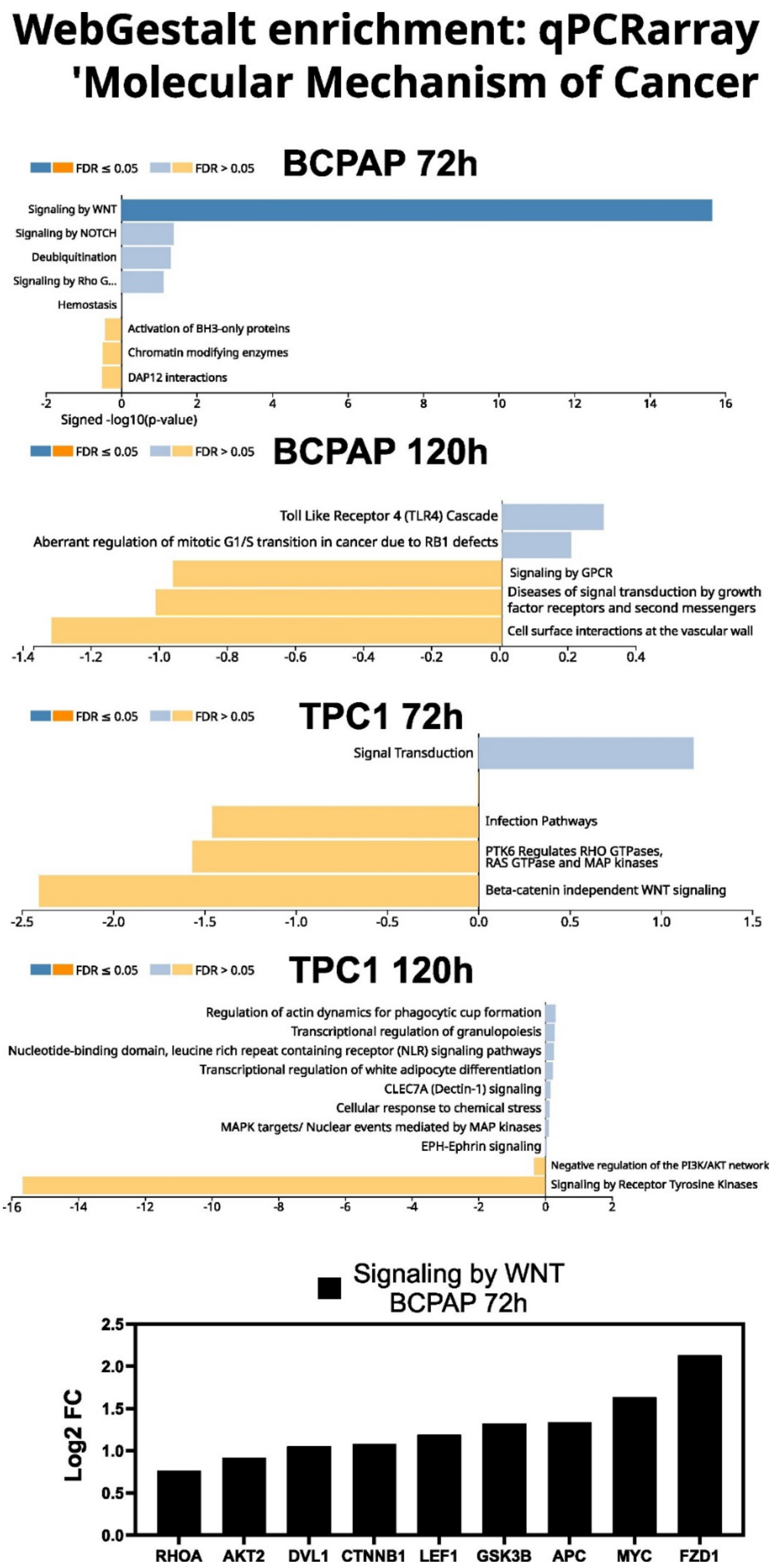

Figure S6. WebGestalt GSEA analysis of the qPCR array 'Mechanisms of Cancer' identified significant regulation of the Signaling by WNT' signaling pathway in BCPAP cells at 72 h. Other pathways, shown in the WebGestalt graphics, showed potential regulation in different treatments but lacked significant False Discovery Rate (FDR) values. Genes regulated in the regulated pathway are shown in the last graph.

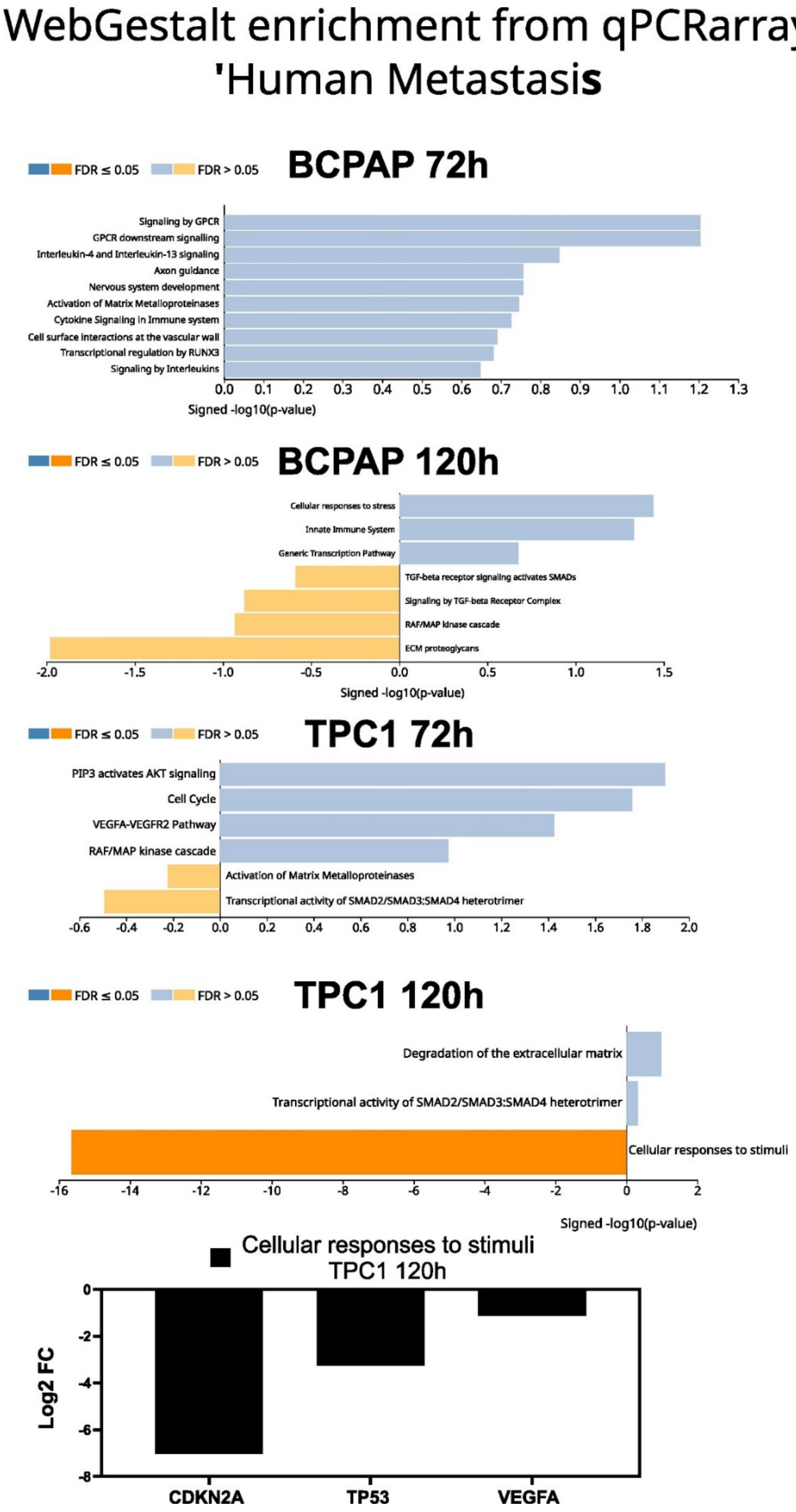

Figure S7. WebGestalt GSEA analysis of the qPCR array 'Human Tumor Metastasis' identified significant regulation of the "Cellular responses to stimuli" pathway in TPC1 cells at 120 hours. Other pathways, shown in the WebGestalt graphics, showed potential regulation in different treatments but lacked significant False Discovery Rate (FDR) values. Genes regulated in the regulated pathway are shown in the last graphic.

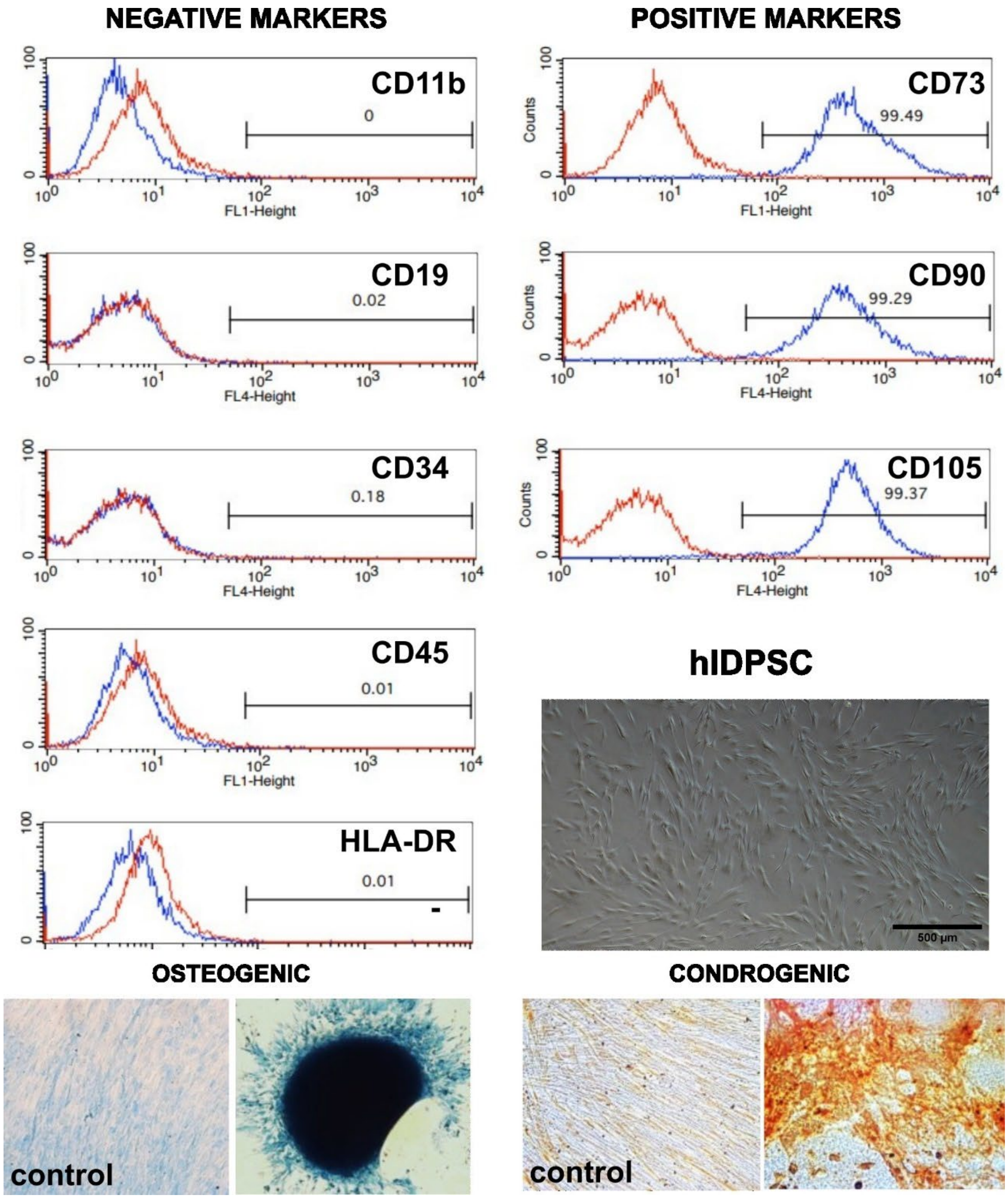

Figure S8. Characterization of hDPSCs by flow cytometry and differentiation assays. Flow cytometry analysis demonstrated the expression of mesenchymal stem cell (MSC) surface markers CD73, CD90, and CD105 (positive markers), and the low/absence of expression of hematopoietic and endothelial cell markers (CD11b, CD19, CD34, CD45, and HLA-DR) (negative markers). Representative images show the capacity of hDPSCs to differentiate into osteogenic (stained with Alizarin Red S) and chondrogenic (stained with Alcian Blue) lineages. Scale bar = 500 μm.

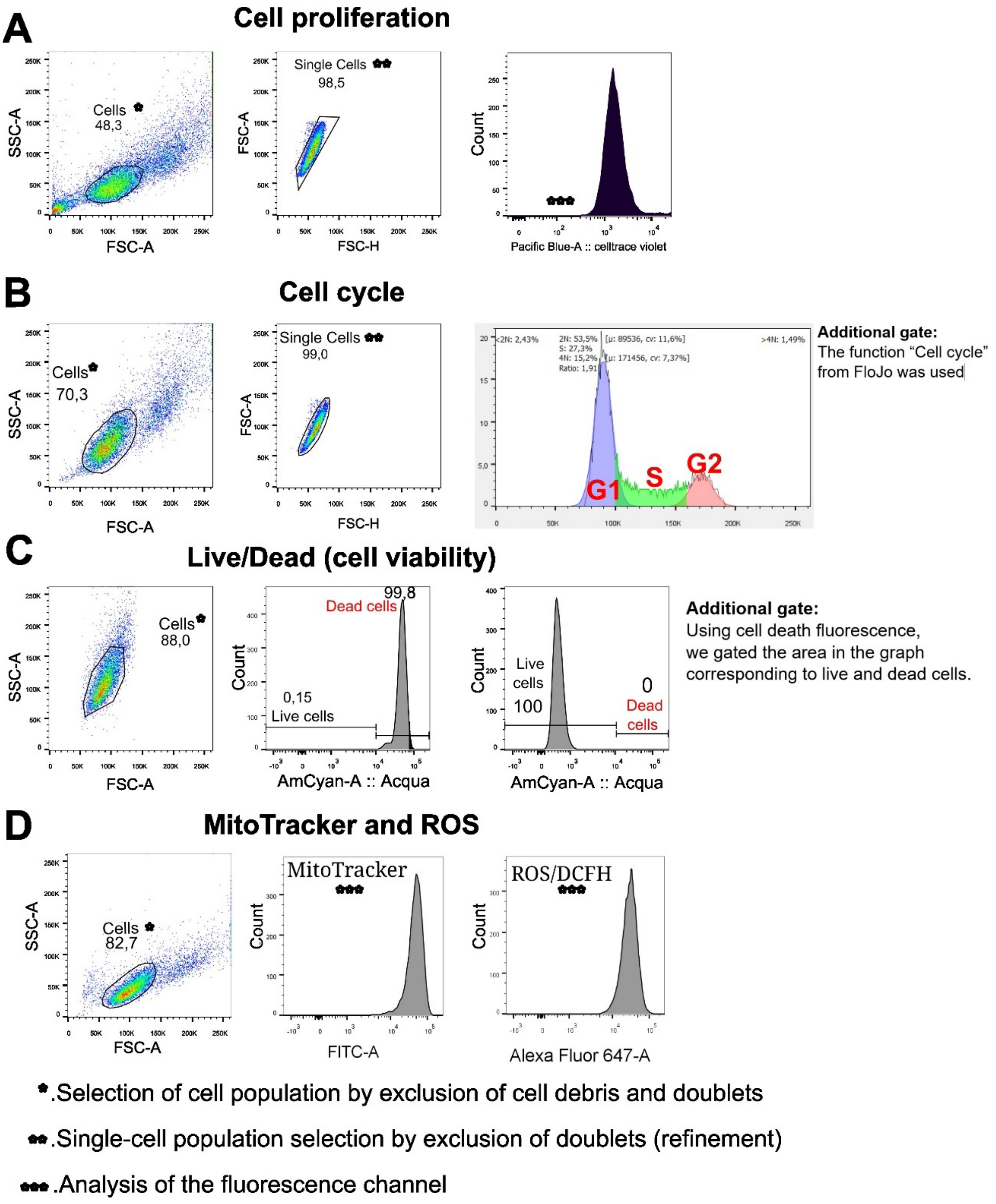

Figure S9. Gate strategies used for flow cytometry analysis.

Table S1. Regulated genes in the qPCR array 'Mechanisms of cancer'. The table displays the Log2Fold change values of cells treated with 50 µg/mL for each regulated gene. N/A represents genes that were not amplified for technical or biological reasons. *Endogenous gene used for normalization

	**Treatments**			
**Genes**	**BCPAP 72 hours**	**BCPAP 120 hours**	**TPC1 72 hours**	**TPC1 120 hours**
18s	-4.58522	-1.73174	-1.04628	14.093135

GAPDH	-1.87142	0.519784	0.135107	0.2662165
*HPRT1**	N/A	N/A	N/A	N/A
*GUSB**	N/A	N/A	N/A	N/A
ABL1	0.535994	-3.34653	0.274024	-0.2322685
AKT1	0.236961	0.444747	0.10272	0.1580245
AKT2	0.91804	-3.12866	N/A	-0.7458835
APC	1.338811	N/A	N/A	N/A
BAX	1.215431	0.421853	-0.63645	0.1164905
BCAR2	0.814927	-3.0773	1.637281	0.4803635
BCL2	N/A	N/A	N/A	N/A
BCL2L1	-0.53409	N/A	0.266082	N/A
BCL2L11	N/A	-2.72418	0.988798	-0.0539385
BID	-1.569853	0.885253	-0.7795	-0.4930855
BRAF	-0.70171	N/A	N/A	-1.4124575
CASP8	N/A	0.802564	0.102964	-0.2059705
CASP9	-0.827458	-0.07421	-0.06061	0.0741105
CCND1	-0.344977	-0.56537	0.316434	-0.1533175
CCND2	N/A	N/A	N/A	N/A
CCND3	1.096645	0.290499	-0.56215	-0.1929465
CCNE1	N/A	0.297407	0.119123	0.2242075
CDC42	N/A	N/A	N/A	N/A
CDH1	N/A	N/A	N/A	N/A
CDK2	0.863884	-0.2601	0.106083	0.0538015
CDK4	0.060292	0.623035	0.244038	0.1719665
CDKN1A	0.321691	0.713247	0.045311	0.1139965
CDKN1B	0.66176	-0.37857	0.007719	-0.5096465
CDKN2A	-1.555572	0.153993	-0.24994	-0.4803775
CDKN2B	N/A	N/A	N/A	N/A
COL1A1	N/A	N/A	N/A	N/A
CRK	-0.711143	0.231969	-0.77694	0.0110025
CTNNB1	1.08127	0.045221	-1.0406	-1.6670505
CYCS	N/A	0.550475	-0.34583	0.8762425
DVL1	1.053435	0.655223	-0.05314	0.4312905
E2F1	-0.166996	0.045055	-0.09774	-0.3867315
EGFR	0.327286	N/A	-0.08546	-0.3226645
ELK1	#VALOR!	0.362035	1.543608	-0.1103815
ERBB2	0.396461	-0.19719	0.1415	-0.7268355
FADD	N/A	1.202973	N/A	N/A
FAS	N/A	-0.55464	0.222899	-0.1579165
FASLG	N/A	N/A	N/A	N/A
FGF2	-0.391467	0.045343	0.078087	-0.0958835
FN1	0.358019	-5.195	0.249147	-0.7132425
FOS	N/A	-0.04851	-0.78689	-0.3000185
FYN	N/A	-0.0982	0.029535	0.0433445
FZD1	2.12743	-0.80405	0.177232	0.5217875

GRB2	1.262089	-0.04908	0.024383	-0.0112455
GSK3B	1.323499	-0.59837	0.243674	-0.3448965
HGF	N/A	N/A	N/A	N/A
HRAS	-0.136368	0.652691	-0.26096	0.1164455
IGF1	N/A	N/A	N/A	N/A
IGF1R	-0.704178	-1.90445	0.491468	-0.5756205
ITGA2B	N/A	0.672097	N/A	-0.4021615
ITGAV	0.185782	-1.17556	0.443952	-0.8388135
ITGB1	0.615449	-0.88093	0.050632	-0.0924245
ITGB3	0.344238	-1.30343	1.13084	-1.2485155
JUN	1.386342	-1.47623	0.640035	0.0968915
KDR	N/A	N/A	N/A	N/A
KIT	N/A	N/A	N/A	N/A
KRAS	-0.596375	N/A	N/A	N/A
LEF1	1.189255	-0.45349	-0.94207	0.3941015
MAP2K1	N/A	-1.08083	-0.67027	-0.5983325
MAP3K5	N/A	-2.01182	-0.00616	-0.3447005
MAPK1	-0.413038	0.067571	0.180902	0.0110755
MAPK14	0.21909	0.212343	0.080105	0.0540205
MAPK3	N/A	0.324503	N/A	0.1229195
MAPK8	N/A	N/A	-0.27631	-0.2900625
MAX	N/A	N/A	N/A	N/A
MDM2	0.172671	-0.9446	0.134414	-0.7084245
MYC	1.633257	-0.21482	-0.06729	-0.0304045
NFKB1	0.264217	0.12334	0.403274	-0.0790425
NFKB2	-0.317918	-0.11667	0.081933	0.1940715
NFKBIA	-1.017983	0.444223	-0.43414	0.3389145
NRAS	-0.998478	0.080369	-0.39771	0.2297425
PIK3CA	N/A	-0.97583	N/A	-0.5574275
PIK3R1	0.771248	N/A	0.46034	-0.1967265
PTEN	N/A	N/A	N/A	N/A
PTK2	-0.361963	-0.46334	0.298372	0.0479125
PTK2B	N/A	0.56198	-0.09322	-0.0367105
RAC1	N/A	-0.20453	-0.04067	-0.0061365
RAF1	0.551396	0.244488	0.16733	0.1348015
RB1	0.165197	-0.14471	0.151281	-0.1266625
RELA	-0.421131	0.318361	0.317667	-0.0478685
RHOA	0.768002	0.247329	-0.7508	0.0298335
SHC1	-2.439093	0.289759	0.168717	-0.1415305
SMAD4	N/A	-0.29854	-0.46357	-0.1875295
SOS1	N/A	N/A	N/A	-1.2210855
SPP1	-0.707603	0.520341	0.300222	0.0622865
SRC	0.124532	-1.10052	0.017601	-0.3286375
TCF3	0.439364	-0.57442	-0.13649	-0.2642855
TGFB1	1.878662	0.516282	0.251484	-0.0593375

TGFBR1	N/A	-2.02958	0.113372	-0.0687765
TGFBR2	0.169637	-0.8599	0.369583	-0.0995215
TP53	0.104233	N/A	0.185871	-2.7758825
VEGFA	0.627677	-0.04569	0.311857	0.0718635
WNT1	N/A	N/A	N/A	N/A

Table S2. Regulated genes in the qPCR array 'Human Tumor Metastasis'. The table displays the Log2Fold change values of cells treated with 50 μg/mL for each regulated gene. N/A represents genes that were not amplified for technical or biological reasons. *Endogenous gene used for normalization

	**Treatments**			
**Genes**	**BCPAP 72 hours**	**BCPAP 120 hours**	**TPC1 72 hours**	**TPC1 120 hours**
18s	N/A	2,3754635	0,9513355	-0,54769
GAPDH	1,429915	0,7186305	0,2895395	0,024997
*HPRT1**	-0,017503	N/A	N/A	N/A
*GUSB**	0,186728	N/A	N/A	N/A
APC	N/A	0,4801295	N/A	-0,18826
BRMS1	0,228158	1,6008185	0,3027135	0,481401
CASP8	1,115222	N/A	-0,472344	0,383315
CCL7	N/A	10,5089945	N/A	N/A
CD44	0,455221	N/A	0,3923625	2,852469
CD82	-3,765482	N/A	0,2388635	1,035798
CDH1	N/A	4,2153615	N/A	N/A
CDKN2A	N/A	N/A	0,5692045	-7,03094
CEACAM1	N/A	0,7232705	N/A	N/A
CTBP1	0,279148	-0,0482175	-0,435121	0,297138
CTNNA1	0,345006	-0,3710665	1,5266745	0,341462
CTSK	-0,360056	N/A	0,2767565	1,112943
CXCL12	N/A	N/A	N/A	N/A
CXCR4	N/A	-0,8065735	N/A	N/A
DAPK1	1,753252	N/A	0,5650635	-0,00748
DCC	N/A	-0,0288045	N/A	N/A
EPHB2	0,925622	-0,2523105	0,5456585	-0,136
ERBB2	-0,043409	0,2074075	0,7771395	0,42077
ETV4	-0,130968	N/A	0,3134475	0,017933
FAT1	N/A	-0,5877225	N/A	N/A
FGF2	0,380878	0,4923075	-0,141526	0,160479
FGFR4	1,03328	-1,0217515	0,4008985	-0,01685
FN1	4,021938	0,2781655	0,7362545	-0,56516
FXYD5	-0,121205	N/A	0,4315615	0,093846
GNRH1	N/A	N/A	0,3775655	-0,02904
HGF	N/A	0,3461975	N/A	N/A
HPSE	0,28933	0,5798215	0,5477695	-0,0987

HRAS	0,018739	0,0477665	-0,228669	0,406859
HTATIP2	0,082443	N/A	0,1142895	0,543841
IGF1	N/A	N/A	N/A	N/A
IL18	N/A	N/A	N/A	N/A
IL1B	N/A	-0,4892345	N/A	N/A
ITGB3	0,94861	0,6933675	1,3302595	-0,40685
KISS1	-0,077022	N/A	1,4866755	0,614344
KISS1R	N/A	-0,2554585	N/A	N/A
KRAS	1,464093	-0,4539205	0,2122285	0,27751
LAMB1	0,795495	N/A	0,3309445	-0,13124
LYPD3	N/A	-0,3037145	N/A	N/A
MCAM	0,344776	-1,0020025	0,1219005	0,058215
MET	1,422326	N/A	0,7772725	-0,4025
MGAT5	N/A	N/A	-0,067714	N/A
MMP1	N/A	N/A	N/A	-0,18627
MMP10	N/A	-0,2868685	N/A	N/A
MMP14	2,692015	0,2050115	0,2876195	-0,63925
MMP2	0,018633	N/A	0,4581955	-0,07064
MMP3	N/A	N/A	N/A	N/A
MMP7	N/A	N/A	N/A	N/A
MMP9	N/A	-8,75E-05	N/A	N/A
MTA1	0,13329	1,6712575	0,1665235	-0,1066
MTA2	N/A	N/A	1,2519445	N/A
MTSS1	N/A	-0,2738255	N/A	-0,4039
MYC	-0,087847	-0,6534645	0,3234965	0,198642
NCAM1	#VALOR!	0,3842625	N/A	0,195697
NF2	-0,272065	0,8280375	0,3696405	0,281412
NME1	0,717612	N/A	-0,124181	0,486054
NR4A3	N/A	N/A	N/A	N/A
PECAM1	N/A	-0,3739795	N/A	N/A
PNN	1,554025	N/A	0,7576395	0,016415
PSCA	N/A	N/A	N/A	N/A
PTEN	N/A	N/A	N/A	N/A
PTGS2	N/A	-0,2646285	N/A	N/A
RB1	0,626499	0,0655255	0,6308795	-0,13708
RBL1	0,890968	-0,4793905	-0,005136	0,178187
RBL2	1,042179	N/A	0,5899295	-0,05652
RET	N/A	1,0713245	N/A	N/A
RHOC	1,07175	0,6376115	0,1403995	0,416496
S100A4	0,9994	N/A	-0,119943	0,200174
SERPINB5	N/A	-0,6322935	N/A	N/A
SERPINE1	0,298224	-1,0250585	0,3697365	0,165734
SET	1,144546	-0,0921245	1,2532585	-0,62862
SMAD2	0,257388	-0,2930945	0,4254055	0,113666
SMAD4	1,118406	0,1822735	0,1511365	0,024144

SNCG	0,23376	N/A	0,3145975	0,175913
SSTR2	N/A	N/A	N/A	N/A
SYK	N/A	N/A	N/A	N/A
TACSTD1	N/A	BCPAP Evs	N/A	N/A
TCF20	1,761215	-0,5974775	1,4305905	-0,11394
TGFB1	-0,247927	-0,0951675	0,5454795	-0,02547
TGFBR2	1,101725	-0,5253545	0,4332315	-0,08688
TIAM1	N/A	-0,9396745	0,4577065	-1,07462
TIMP1	-0,178855	0,4387215	0,5085915	-0,07213
TIMP2	0,492485	0,3337915	0,2894975	-0,07627
TIMP4	N/A	N/A	N/A	N/A
TMPRSS4	N/A	N/A	N/A	N/A
TNFSF10	N/A	-0,2637045	N/A	0,295745
TP53	-0,151108	3,6030385	2,2258285	-3,24215
TPBG	-0,158545	-0,5930045	0,1112775	-0,68391
TSHR	N/A	N/A	N/A	N/A
TWIST1	N/A	0,0319375	0,1360965	N/A
VEGFA	1,053964	-0,0718245	-0,148199	-1,1167
VEGFC	0,155278	0,0710985	0,2976015	0,289036
WISP	N/A	N/A	N/A	N/A
